# The public health value of vaccines beyond efficacy: methods, measures and outcomes

**DOI:** 10.1186/s12916-017-0911-8

**Published:** 2017-07-26

**Authors:** A. Wilder-Smith, I. Longini, P. L. Zuber, T. Bärnighausen, W. J. Edmunds, N. Dean, V. Masserey Spicher, M. R. Benissa, B. D. Gessner

**Affiliations:** 1 Lee Kong Chian School of Medicine, Nanyang Technological University, Singapore, Singapore; 2 Institute of Public Health, University of Heidelberg, Heidelberg, Germany; 3 London School of Hygiene and Tropical Medicine, London, UK; 4 University of Florida, Gainesville, FL USA; 5 World Health Organization, Geneva, Switzerland; 6 Federal Office of Public Health, Bern, Switzerland; 7 Institute of Global Health, University of Geneva, Geneva, Switzerland; 8 Agence de Médecine Preventive (AMP), Paris, France

**Keywords:** Vaccine efficacy, Effectiveness, Overall effectiveness, Vaccine-preventable disease incidence, Public health impact, Dynamic modelling, Cluster randomised controlled trial, Pre-licensure, Post-licensure, Quasi-experiments

## Abstract

**Background:**

Assessments of vaccine efficacy and safety capture only the minimum information needed for regulatory approval, rather than the full public health value of vaccines. Vaccine efficacy provides a measure of proportionate disease reduction, is usually limited to etiologically confirmed disease, and focuses on the direct protection of the vaccinated individual. Herein, we propose a broader scope of methods, measures and outcomes to evaluate the effectiveness and public health impact to be considered for evidence-informed policymaking in both pre- and post-licensure stages.

**Discussion:**

Pre-licensure: Regulatory concerns dictate an individually randomised clinical trial. However, some circumstances (such as the West African Ebola epidemic) may require novel designs that could be considered valid for licensure by regulatory agencies. In addition, protocol-defined analytic plans for these studies should include clinical as well as etiologically confirmed endpoints (e.g. all cause hospitalisations, pneumonias, acute gastroenteritis and others as appropriate to the vaccine target), and should include vaccine-preventable disease incidence and ‘number needed to vaccinate’ as outcomes.

Post-licensure: There is a central role for phase IV cluster randomised clinical trials that allows for estimation of population-level vaccine impact, including indirect, total and overall effects. Dynamic models should be prioritised over static models as the constant force of infection assumed in static models will usually underestimate the effectiveness and cost-effectiveness of the immunisation programme by underestimating indirect effects. The economic impact of vaccinations should incorporate health and non-health benefits of vaccination in both the vaccinated and unvaccinated populations, thus allowing for estimation of the net social value of vaccination.

**Conclusions:**

The full benefits of vaccination reach beyond direct prevention of etiologically confirmed disease and often extend across the life course of a vaccinated person, prevent outcomes in the wider community, stabilise health systems, promote health equity, and benefit local and national economies. The degree to which vaccinations provide broad public health benefits is stronger than for other preventive and curative interventions.

## Background

**Fig. 1:**
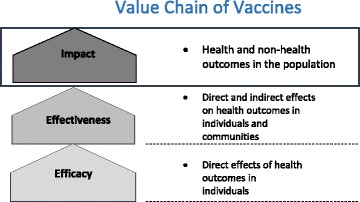
Value chain of vaccines

Evidence of vaccine efficacy and safety in the directly vaccinated individual are the primary factors evaluated when determining licensure. Regulatory decisions consequently focus on benefit–risk ratios, as measured only by vaccine efficacy and safety. However, measures of vaccine efficacy and safety data capture only the minimum information needed for regulatory approval, and do not capture the full public health value of vaccines. Vaccine efficacy derived from pre-licensure phase III trials only provides a measure of proportionate disease reduction, is usually limited to etiologically confirmed disease, and focuses on direct protection in the vaccinated individual. As individually randomised trials (iRCT) do not allow a full assessment of indirect vaccine protection, relying only on them will underestimate the public health value of vaccines that have high indirect effects. Thus, all aspects of the public health value of vaccines should be evaluated and incorporated into public health decision-making. The full public health impact of vaccinations should consider health and non-health benefits of vaccination in both vaccinated and unvaccinated populations. Indeed, the value chain goes beyond efficacy and effectiveness to include the broader public health impact (Fig. 1). Although safety is equally important in policymaking, methods for monitoring the safety profile of vaccines have been addressed in other recent publications [1, 2] and will not be re-addressed in this paper.

To expand regulatory and policy discussions, the Fondation Merieux organised a conference in June 2015 entitled Beyond Efficacy: The Full Public Health Impact of Vaccines in Addition to Efficacy Measures in Trials. As a result, a series of recommendations have been published [3]. During the Geneva Health Forum 2016, we further developed these recommendations into a methodologically rigorous theoretical framework. Herein, we propose a broader scope of methods, measures and outcomes to evaluate the effectiveness and public health impact of vaccines to be considered for evidence-informed policymaking at both the pre- and post-licensure level.

## Disease and health outcomes

### Definitions of efficacy and effectiveness

‘Efficacy’ is usually defined as the performance of an intervention under ideal and controlled circumstances, whereas ‘effectiveness’ refers to its performance under ‘real-world’ conditions. For the purpose of this manuscript, we will apply a more rigorous mathematical definition of efficacy as the proportionate reduction of the incidence of the target infection in vaccinated subjects compared to controls [4], equalling one minus the hazard or risk ratio. For vaccine efficacy, we require that vaccinated and unvaccinated people have the same exposure risk to the target etiology, a condition best achieved through randomised vaccine allocation.

**Fig. 2:**
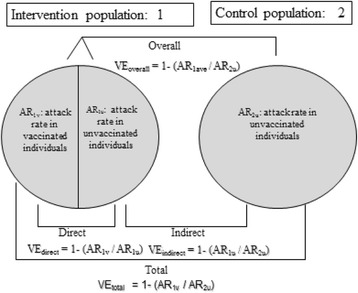
Types of vaccine effectiveness, as developed by Halloran et al. [34]. Cluster 1 has a fraction of the population vaccinated, while cluster 2 has no person vaccinated. The u and v indices designate vaccinated and unvaccinated people, respectively. Direct effectiveness compares the attack rate (AR) (or some other rate measure, e.g. incidence) of vaccinated to unvaccinated people within a cluster, as in cluster 1. Indirect effectiveness compares the AR in unvaccinated people in the partially vaccinated cluster 1 to the AR in an unvaccinated cluster 2. Total effectiveness compares the AR of vaccinated people in cluster 1 to the AR in cluster 2. Finally, overall effectiveness compares the AR among all people in cluster 1 (i.e. vaccinated and unvaccinated) to the AR among all people in cluster 2

Vaccine effectiveness is defined as the reduction of the incidence for those receiving the vaccine intervention in relation to direct, indirect, total and overall effectiveness [5] (Fig. 2). As Fig. 2 indicates, indirect effectiveness measures the reduction in incidence of unvaccinated people in a population targeted for vaccination with a varying level of immunisation coverage compared to that for unvaccinated people in a totally unvaccinated population.

### Methods to measure indirect effects in pre-licensure trials

An iRCT is the traditional method to measure vaccine efficacy, evaluating a vaccine against a placebo or active comparator with pre-specified endpoints to measure the treatment effect size [6]. Such a trial design provides data on direct vaccine efficacy but does not provide information on indirect, total and overall effects.

In addition to the lack of information on non-direct vaccine effects, licensing trials have other limitations. For rare outcomes, such as death, iRCTs may fail to recruit sufficient sample sizes. For long-term outcomes, such as neurologic sequelae of meningitis, licensing trials may have insufficiently long follow-up periods. Etiologies driven by largely unpredictable outbreaks, such as cholera, dengue, serogroup A meningococcus, Ebola and others, may assume an importance well beyond their disease burden since they can cause health system disruption, political instability and acute economic loss. Licensing trials based on an iRCT design may be difficult to implement during an outbreak setting, are not optimal for demonstrating a vaccine’s ability to prevent outbreaks rather than individual cases of disease, and do not provide information on a vaccine’s ability to prevent health system disruption resulting from an outbreak or epidemic. All of these issues may contribute to decision-makers failing to appreciate the full benefits of a vaccine, and thus delay its introduction.

In some circumstances, licensing trials may need to rely on cluster-randomised clinical trial (cRCT) designs. The advantage of cRCTs is that they support estimation of population-level vaccine effects, including indirect, total and overall effects (Fig. 2). Specifically, in a cRCT, the main outcome is total vaccine effect, which includes both direct and indirect effects. Indirect effects can be measured independently by comparing unvaccinated persons in intervention clusters (usually due to non-compliance) to unvaccinated persons in control clusters. If intervention clusters include individual-level randomisation, one could retain the benefits of an iRCT by having a true measure of vaccine efficacy as defined previously. However, in this circumstance, indirect benefits are fixed at the level seen by the vaccine coverage dictated by the randomisation scheme and may not reflect real-world experience. Moreover, having an iRCT nested within a cRCT presents considerable logistic challenges.

An example of a cRCT design during an outbreak setting was the ring vaccination trial of the replication-competent vesicular stomatitis virus-based vaccine Ebola vaccine candidate in Guinea, West Africa, during the Ebola outbreak [5, 7]. The unit of randomisation was contacts of an Ebola case and their contacts (i.e. contacts of contacts). This approach allowed for faster recruitment at a time when Ebola incident cases were declining and the geographic location of the next cluster of cases was unpredictable. The cluster in a cRCT could be a household, neighbourhood, village, district or a network of contacts. Despite this flexibility, several factors must be considered. Clusters should be selected to reduce the chance of movement of people and vectors between clusters. A cRCT design could require a larger sample size, but this may be counter-balanced by a higher event rate if the cluster is a highly targeted at-risk population. Unless the intervention arm is individually randomised, a cRCT will not provide an unbiased measure of direct vaccine efficacy. Lastly, cRCTs may be more difficult to implement and thus cost more, take longer or increase the risk of implementation failure. However, for many vaccines where a high indirect effect is anticipated, such disadvantages are far outweighed by the added information provided through cRCTs. We suggest that regulatory authorities should consider allowing cRCTs to become an acceptable additional pathway for licensure when iRCTs are impractical or where high indirect effects are anticipated.

### Post-licensure evaluation of vaccines

Countries that introduce vaccines post-licensure should plan to continue surveillance of the targeted disease to allow evaluation of vaccine impact. Vaccine efficacy from pre-licensure studies is often erroneously used as a surrogate for predicting reductions in disease burden, but inaccurately predicts impact for the reasons previously discussed. Higher than expected post-licensure reductions will occur where a vaccine has a high indirect effect. An additional issue can arise when burden reduction estimates are based on a surrogate. For example, for pneumococcal conjugate vaccine (PCV), vaccine efficacy against non-bacteremic pneumonia was based on vaccine efficacy for invasive pneumococcal disease. If the former is substantially less than the latter, then disease burden estimates would have been underestimated. Since PCV has large indirect effects through reduction of carriage, vaccine serotype pneumococcal pneumonia would eventually be eliminated even with a lower vaccine efficacy.

Lower than expected post-licensure reductions in disease burden may indicate problems with vaccine delivery, low immunisation coverage (due to programmatic problems or vaccine hesitancy), cold chain limitations or reflect different vaccine schedules compared to those used in pre-licensure trials. In theory, lower than expected reductions may occur if lower efficacy or effectiveness is present in some epidemiological settings, such as the hypothesis that observed lower rotavirus vaccine efficacy in some settings occurred due to differences in gut flora. Practically, however, we are not aware of circumstances in which licensing trials using an iRCT design overestimated disease burden reductions. For the rotavirus vaccine trials, settings in which vaccine efficacy was lower had a higher vaccine-preventable disease incidence (VPDI) due to much larger background incidence rates. Regardless, documenting and publicising vaccine impact under real-life settings can provide important public and political support for routine immunisation programs. Post-licensure surveillance, including surveillance for adverse events following immunisations, is also important to identify rare adverse events or unexpected effects. Robust vaccine impact data can help to counter anti-vaccine messaging.

#### Methods

Post-licensure vaccine effectiveness trials are population-specific trials that focus on estimating the public health impact of the vaccine in a particular setting under real world conditions [4]. Most post-licensure studies are observational given the ethical issues of withholding a licensed vaccine from a control group. Under some circumstances, however, randomised studies may be conducted. For example, if disease burden is unknown (and thus a vaccine would not be introduced), a vaccine can be used in a probe study approach [8], as occurred during a *Haemophilus influenzae* type b (Hib) vaccine trial in Indonesia [9]. Vaccine introduction may also need to be implemented in stages due to programmatic, product or financial limitations that would allow for a stepped wedge design, although, for a variety of reasons, this design has almost never been used in a vaccine trial [10, 11].

A last justification for a randomised trial using a cluster design in the post-licensure phase may be to demonstrate vaccine performance (including vaccine efficacy and impact) in a resource-poor setting where many health priorities compete for scarce health sector funding. In this case, cluster randomisation is particularly important since the goal is to provide as accurate an estimate as possible of vaccine-associated reductions in adverse health outcomes. One example of this was a phase IV double-blind, placebo-controlled trial of a two-dose regimen of bivalent killed whole-cell oral cholera vaccine over 5 years in a slum area of Kolkata, India [12]. A second example is a phase IV trial for a single dose of the Vi polysaccharide typhoid vaccine in slum-dwelling residents of Kolkata, India [13].

Mathematical models can be used to extrapolate from the results of clinical trials to estimate the impact of vaccination programmes. The models that are used for this purpose can be classified as dynamic or static. Dynamic transmission models (often shortened to ‘dynamic models’) are able to capture the direct and indirect vaccine effects by assuming that the probability of a susceptible individual becoming infected at any one point in time (the force of infection) is related to the number of infectious individuals in the population. If this changes (for instance, vaccination would be expected to reduce the number of infectious individuals), then the model recalculates the force of infection. Thus, the remaining susceptible people experience a reduced risk of infection through indirect protection [14, 15]. These assumptions closely mirror the real-world epidemiology of most vaccine-preventable diseases. Static models, by contrast, do not recalculate the force of infection – it remains at a fixed level (usually the pre-vaccination level) – and therefore the remaining susceptible individuals in the modelled population do not experience any indirect protection as a result of the vaccination programme. By omitting indirect effects, static models underestimate immunisation programme impact. Despite this, static models remain widespread. Indeed, most economic analyses of vaccination programmes employ these methods [16] and therefore underestimate the cost-effectiveness of vaccination programmes. There are other, potentially important, effects of vaccination programmes that are not captured by static models, and that can be predicted by appropriately parameterised dynamic models. These include increases in the average age at infection following infant immunisation (which may have important public health consequences if the risk of serious outcomes of infection increases or decreases with age as is the case with rubella and malaria, respectively) [15], increasing gaps between epidemics, and replacement of vaccine-targeted serotypes with non-vaccine types (as has been demonstrated with PCVs that target a relatively limited repertoire of the more than 90 pneumococcal serotypes). These different effects, which are often vaccine-specific, require the development of specific dynamic models.

#### Measures

We propose that the measures of VPDI, the number needed to vaccinate (NNV) and total cases prevented should be used in a more systematic manner for all vaccines [17, 18]. VPDI has several synonyms, including vaccine-attributable risk and vaccine-attributable rate reduction. It is the incidence of a given disease syndrome preventable by vaccine in a given context [19], and is defined as “*outcome incidence in an unvaccinated population X vaccine efficacy*” [8], and thus incorporates both vaccine efficacy and the underlying burden of disease. This is mathematically equivalent to the incidence in the control group minus the incidence in the intervention group. VPDI derived from a clinical trial is reported as cases per 100,000 vaccinated persons per year for the duration of the trial. In principle, and as indicated above, VPDI is best calculated from cRCTs as this allows incorporation of the vaccine’s ability to prevent disease through both direct and indirect mechanisms and, in this case, it is the overall incidence reduction in the vaccinated population that is achievable with vaccine. By contrast, VPDI calculated from an iRCT gives only the reduction in incidence achievable from direct immunisation of individuals. Community randomised trials, however, are rare. For example, studies of PCV [9] and rotavirus [17, 18] vaccines used iRCT designs to assess VPDI against clinical outcomes; these studies likely underestimated VPDI since rotavirus vaccine and PCV provide indirect protection. Studies of dengue [20] and the RTS,S malaria [10] vaccines similarly used an iRCT design, but the consequence of this is unknown since vaccination against vector-borne diseases affecting the entire population may provide minimal transmission reductions when the vaccine target age range is highly constricted. An additional issue is that iRCTs and cRCTs both usually target a limited age range even if indirect benefits may accrue to other persons. For example, PCVs may provide most of their benefit via indirect protection of unvaccinated older persons.

The NNV is often used as a metric of the value of vaccination programmes, and can also be used for cost effectiveness studies. NNV is a measure to quantify the number of people that need to be vaccinated, or the number of vaccine doses that need to be used, to prevent one occurrence of a target health outcome [21]. Unlike VPDI, NNV is not a rate but instead the overall number of cases prevented for a given number of persons vaccinated, and thus incorporates the length of the trial or, outside of a trial, the duration of immunity. Consequently, if VPDI is reported as cases per 100,000 vaccinated persons per year, NNV is calculated as 100,000 divided by VPDI divided by length of study/immune duration.

While VPDI and NNV can be calculated for etiologically confirmed outcomes, as public health measures, these metrics have more utility when calculated for clinical outcomes as this adjusts for the inevitable failure to confirm all prevented outcomes. Although less specific outcomes lead to lower vaccine efficacy, the baseline incidence for less specific outcomes is often much higher, leading to higher and more accurate VPDI estimates. For example, during PCV trials in The Gambia [9] and South Africa [22], the vaccine prevented approximately 4- to 5-fold more clinical pneumonia than vaccine serotype invasive pneumococcal disease. A trial of Hib conjugate vaccine in Indonesia used a community randomised design to assess VPDI for clinical pneumonia and suspected meningitis, and found a VPDI for all clinical meningitis 10-fold higher than that for confirmed Hib meningitis [23]. Similar effects are seen in developed countries and emphasise the difficulty in accurately confirming etiology for all or even most cases in which an organism forms part of the causal chain. For example, in Finland, rotavirus VPDI was over twice as high for all-cause compared to confirmed rotavirus acute gastroenteritis [24].

Another advantage of focusing VPDI and NNV on clinical outcomes is that clinical outcomes are usually of greater public health importance and allow for more accurate comparisons between vaccines. For example, public health officials have a greater interest in preventing hospitalisation for pneumonia than in preventing pneumonia through a serotype invasive pneumococcal disease vaccine. When valuing a new vaccine such as for RTS,S malaria or dengue, it may be more sensible, from a public health perspective, to compare VPDI or NNV against severe fever (or severe fever hospitalisations) rather than VPDI or NNV for PCV or Hib vaccine impact against severe pneumonia or rotavirus vaccine against severe gastroenteritis [7].

Nevertheless, VPDI and NNV have some limitations. Because these metrics incorporate baseline disease incidence, they depend on the local epidemiological context and thus require an appreciation of local epidemiological nuances when extrapolating from one setting to another. However, this may also represent an advantage since it emphasises that decision-making around vaccines should reflect not just the degree to which the vaccine works against the target etiology but also how much disease can potentially be prevented.

### Outcomes

Outcomes should be those that are feasible, ethical and of public health importance. Mortality is of the highest public health importance. However, too few cases may occur to make this a feasible outcome in all but the largest trials. Additionally, there is a moral imperative to provide a certain standard of healthcare to study participants, decreasing mortality in trials even further. For example, the RTS,S malaria vaccine trial was unable to assess impact against malaria-related deaths because so few subjects died irrelevant of their vaccination status [25]. Disease syndromes often have higher public health relevance for etiologically confirmed disease and, therefore, studies of PCV assessed impact against severe pneumonia rather than invasive pneumococcal disease as a primary outcome. In some instances, an outcome can approximate both an etiology and a syndrome, for example, in measles, malaria and dengue. In this case, the primary advantage of using non-specific outcomes (such as fever, or fever and rash) would be to assess the degree to which diagnostic testing missed cases, for example, through failure to suspect disease, failure to collect a diagnostic specimen, laboratory error or imperfect test sensitivity.

Usually, severe disease is the optimal health outcome to measure impact, and for some etiologies this is codified. However, severe disease case definitions often do not exist. For example, no clinical trial case definition of severe pneumonia currently exists. Studies have used hospitalisation as a proxy, even though this is a utilisation and not a severity measure.

Table 1 summarises the pre- and post-licensure methods, measures and outcomes for health outcomes.

**Table 1:** Public Health impact of vaccines related to health outcomes

Health-related evaluations of impact of vaccines	Conventional evaluations	Additional ‘broader’ evaluations
Methods	*Pre-licensure* Individually randomised controlled Phase III trials *Post-licensure* Total and indirect effectiveness studies Static modelling (assuming constant force of infection)	*Pre-licensure* Cluster randomised controlled trials Dynamic models *Post-licensure* Cluster randomised trials Probe studies Pre/post evaluations Observational controlled studies
Measures	Efficacy and effectiveness	Vaccine preventable disease incidence Number needed to vaccinate
Outcomes	Morbidity and mortality at individual level	All-cause mortality Under 5 mortality Non-etiologically confirmed clinical syndromes

## Beyond health outcomes

The full public health impact of vaccinations should incorporate both the health and non-health benefits of vaccination in both the vaccinated and unvaccinated populations, i.e. including spillover effects (or indirect effects or ‘externalities’) regarding all outcomes that matter to the population or to policymakers. The inclusion of internal and external non-health benefits in public health impact is important since they allow movement from standard cost-effectiveness analyses (with health effects and direct and indirect healthcare expenditure) to the estimation of the net social value of vaccination [26]. The net social value should ideally capture internal and external vaccination effects on outcomes such as educational attainment, employment, income and social functioning – if those effects matter to populations or policymakers. While such effects should ideally be included in the estimation of public health impacts of all health interventions, these effects are particularly important for vaccinations because disease prevention in childhood often affects entire life trajectories. For instance, a child’s economic future will be very different if she becomes blind as a consequence of a measles infection; however, the economic and social effects of blindness due to measles infection will not be fully captured in standard efficacy, effectiveness and cost-effectiveness analyses.

### Methods

The causal effects of vaccinations on non-health outcomes, such as education and income, can sometimes be established in an iRCT. For instance, by revisiting children who participated in iRCTs years after completion of the trial, the causal effect of the vaccination on long-term cognitive development and income can be determined (if the control group did not receive the vaccination after completion of the trial). However, in many cases, it will be difficult to re-visit the trial subjects many years later or subjects assigned to the control trial group will have received the vaccination. Therefore, we also need to use quasi-experiments to establish the long-term effects of vaccinations on non-health outcomes in both those receiving and those not receiving a vaccination. Quasi-experiments utilise quasi-random assignment to intervention and control groups that results from policy, clinical practice or ‘nature’. In quasi-experiments, the unconfoundedness assumption required in non-experimental studies for causal inference is replaced with other, often substantially weaker, assumptions. Examples for such assumptions include the exclusion restriction of instrumental variable analysis, the continuity assumption of regression discontinuity analysis, and the parallel trends assumption of difference-in-differences analysis [27]. Quasi-experiments have the advantage over many RCTs that they typically generate causal evidence with a high degree of external validity since they rely on routinely collected outcome data and use real-life vaccination policies and practices as exposure, i.e. they avoid the artificial contexts that are often created by the selection criteria and processes in RCTs. Quasi-experiments can often be carried out at low cost, using routinely collected and retrospective data. They further allow long-term follow-up of important health and non-health outcomes of vaccinations in general populations [28], which is useful for extended cost-effectiveness analysis and to estimate the full public health impact and net social value of vaccinations. While quasi-experiments are still relatively rare in vaccination studies, they are being increasingly utilised in this field [29–31].

### Measures and outcomes

Depending on the etiology and the reasons for using a vaccine, outcomes other than those related to health should be documented. As vaccination confers benefits that are often neglected by traditional economic evaluations, thereby resulting in sizeable undervaluation of vaccination programmes [32], we propose moving from a narrow economic focus, such as care-related productivity gains and healthcare cost savings, to a broader range of economic outcomes (Table 2). These additional outcomes could include the vaccine programme’s impact on spending on outbreak control, tourism (in particular for vaccines that prevent or mitigate outbreaks), economic productivity, and community health externalities in terms of improved health outcomes for unvaccinated community members [33].

**Table 2:** Public health impact of vaccines related to non-health outcomes

Non-health related evaluations of impact of vaccines	Traditional evaluations	Additional ‘broader’ evaluations
Methods	Cost-effectiveness analysis, informed by clinical trials and costing studies	Extended cost effectiveness analysis, informed by quasi-experiments
Measures and outcomes	Healthcare cost savings Care-related productivity gains Health gains	Outcome- and behaviour-related productivity gains Community health externalities (e.g. improved health outcomes in unvaccinated community members) Improved economic outcomes at the household and societal levels Reduction in tourism loss Averted outbreaks Reduced health system disruption and impact on other diseases

## Conclusions

At a pre-licensure level, we have outlined the limitations of relying on efficacy data derived from assessment of etiologically confirmed outcomes and the limitations of the requirement for iRCTs as the only pathway to licensure. Conventional pre-licensure trials exacerbate uncertainties by focusing on measurement of vaccine efficacy under idealised conditions [4]. iRCTs will hence often grossly underestimate total vaccine impact by omitting the indirect effects of vaccines and secondary outcomes beyond morbidity and mortality at the individual level. We suggest that, where iRCTs are not feasible, regulatory authorities should consider allowing cRCTs to become an acceptable additional pathway for licensure and reduce the divide between pre- and post-licensure evaluations. While we acknowledge that regulatory concerns often dictate an iRCT as the preferred design for phase IIb or III vaccine efficacy trials, certain circumstances, such as the Ebola epidemic or vaccines with anticipated high indirect effects, do require designs such as the cRCT, which should be considered valid for licensure by regulatory agencies. Furthermore, analytic plan reporting for pre-licensure phase III pivotal trials should always include incidence rate reductions (VPDI) and NNV. Additionally, the analytic plans should also ideally include assessment of vaccine impact on clinical as well as etiologically confirmed outcomes (e.g. all cause hospitalisations, pneumonias, acute gastroenteritis and others, as appropriate to the vaccine target). Finally, dynamic mathematical models can be used to extrapolate from the results of pre-licensure clinical trials to estimate the impact of vaccination programmes.

At the post-licensure level, we have entered an era of vaccine evaluation where all aspects of the public health value of vaccines beyond efficacy should be assessed [3]. This is particularly important for vaccines with moderate efficacy such as the RTS,S/AS01 malaria vaccine, CYD-TDV dengue vaccine against non-hospitalised dengue cases, and rotavirus vaccine in epidemiological settings with a high disease burden. Phase IV trials that focus on public health outcomes should ideally use a cRCT design that allows for estimation of population-level vaccine impact, including indirect, total and overall effects. These measures provide a more complete indication of the public health value of vaccines than just direct protection of individuals and report total vaccine impact on outcome incidence rates. As these studies are not required for licensing purposes, vaccine manufacturers often do not fund them, unless they are required to do so as a post-authorisation commitment or for label updates (e.g. seeking a new indication). Hence, such studies are often funded by public health agencies or external organisations such as The Bill & Melinda Gates Foundation or Gavi. Nevertheless, the industry has been known to fund such studies, depending on the alignment of interests and resources; for example, in studies where they think their product is underutilised because its value has been underestimated in pre-licensure studies, and in these cases may try to perform studies that can be generalised to a broader group of settings. Public health agencies may fund studies to determine if a vaccine is under- or overvalued in their population; external agencies such as Gavi usually support such studies in resource-limited settings.

Modelling is also a valuable tool, but dynamic models should be prioritised over static models as the constant force of infection assumed in static models will usually underestimate the effectiveness and cost-effectiveness of the immunisation programme by underestimating the indirect effects [15]. Post-licensure quasi-experiments allow long-term follow-up of important non-health outcomes of vaccinations in general populations in order to estimate the full public health impact and net social value of vaccinations.

Policymakers should base their decision-making on a broader range of measures and outcomes to more fully assess a vaccine’s public health impact at the individual and societal levels. We suggest expanding the traditionally narrow focus (on direct reduction in morbidity/mortality, care-related productivity gains and healthcare cost savings) to a larger range of measures and outcomes. The full public health impact of vaccinations should incorporate both the health and non-health benefits in vaccinated and unvaccinated populations, i.e. include indirect benefits and spillover effects (or ‘externalities’) regarding all outcomes. Although such externalities and indirect effects are important for all types of health interventions, the a priori case for broader effects is stronger for vaccinations than for any other health intervention [29].

## References

[1] Council for International Organizations of Medical Sciences (2017). CIOMS Guide to Active Vaccine Safety Surveillance: Report of CIOMS Working Group on Vaccine Safety.

[2] Andrews N (2012). Epidemiological designs for vaccine safety assessment: methods and pitfalls. Biologicals.

[3] Saadatian-Elahi M, Horstick O, Breiman RF, Gessner BD, Gubler DJ, Louis J, Parashar UD, Tapia R, Picot V, Zinsou JA (2016). Beyond efficacy: the full public health impact of vaccines. Vaccine.

[4] Clemens J, Brenner R, Rao M, Tafari N, Lowe C (1996). Evaluating new vaccines for developing countries. Efficacy or effectiveness?. JAMA.

[5] Henao-Restrepo AM, Longini IM, Egger M, Dean NE, Edmunds WJ, Camacho A, Carroll MW, Doumbia M, Draguez B, Duraffour S (2015). Efficacy and effectiveness of an rVSV-vectored vaccine expressing Ebola surface glycoprotein: interim results from the Guinea ring vaccination cluster-randomised trial. Lancet.

[6] Russek-Cohen E, Rubin D, Price D, Sun W, Cox E, Borio L (2016). A US Food and Drug Administration perspective on evaluating medical products for Ebola. Clin Trials.

[7] Ebola ça Suffit Ring Vaccination Trial Consortium (2015). The ring vaccination trial: a novel cluster randomised controlled trial design to evaluate vaccine efficacy and effectiveness during outbreaks, with special reference to Ebola. BMJ.

[8] Feikin DR, Scott JA, Gessner BD (2014). Use of vaccines as probes to define disease burden. Lancet.

[9] Cutts FT, Zaman SM, Enwere G, Jaffar S, Levine OS, Okoko JB, Oluwalana C, Vaughan A, Obaro SK, Leach A (2005). Efficacy of nine-valent pneumococcal conjugate vaccine against pneumonia and invasive pneumococcal disease in The Gambia: randomised, double-blind, placebo-controlled trial. Lancet.

[10] Mdege ND, Man MS, Taylor Nee Brown CA, Torgerson DJ (2011). Systematic review of stepped wedge cluster randomized trials shows that design is particularly used to evaluate interventions during routine implementation. J Clin Epidemiol.

[11] Beard E, Lewis JJ, Copas A, Davey C, Osrin D, Baio G, Thompson JA, Fielding KL, Omar RZ, Ononge S (2015). Stepped wedge randomised controlled trials: systematic review of studies published between 2010 and 2014. Trials.

[12] Bhattacharya SK, Sur D, Ali M, Kanungo S, You YA, Manna B, Sah B, Niyogi SK, Park JK, Sarkar B (2013). 5 year efficacy of a bivalent killed whole-cell oral cholera vaccine in Kolkata, India: a cluster-randomised, double-blind, placebo-controlled trial. Lancet Infect Dis.

[13] Sur D, Ochiai RL, Bhattacharya SK, Ganguly NK, Ali M, Manna B, Dutta S, Donner A, Kanungo S, Park JK (2009). A cluster-randomized effectiveness trial of Vi typhoid vaccine in India. N Engl J Med.

[14] Pitman R, Fisman D, Zaric GS, Postma M, Kretzschmar M, Edmunds J, Brisson M, Force I-SMGRPT (2012). Dynamic transmission modeling: a report of the ISPOR-SMDM Modeling Good Research Practices Task Force Working Group-5. Med Decis Making.

[15] Edmunds WJ, Medley GF, Nokes DJ (1999). Evaluating the cost-effectiveness of vaccination programmes: a dynamic perspective. Stat Med.

[16] Goldie SJ, O'Shea M, Campos NG, Diaz M, Sweet S, Kim SY (2008). Health and economic outcomes of HPV 16,18 vaccination in 72 GAVI-eligible countries. Vaccine.

[17] Feikin DR, Laserson KF, Ojwando J, Nyambane G, Ssempijja V, Audi A, Nyakundi D, Oyieko J, Dallas MJ, Ciarlet M (2012). Efficacy of pentavalent rotavirus vaccine in a high HIV prevalence population in Kenya. Vaccine.

[18] Madhi SA, Cunliffe NA, Steele D, Witte D, Kirsten M, Louw C, Ngwira B, Victor JC, Gillard PH, Cheuvart BB (2010). Effect of human rotavirus vaccine on severe diarrhea in African infants. N Engl J Med.

[19] Gessner BD, Feikin DR (2014). Vaccine preventable disease incidence as a complement to vaccine efficacy for setting vaccine policy. Vaccine.

[20] Gessner BD, Wilder-Smith A (2016). Estimating the public health importance of the CYD-tetravalent dengue vaccine: vaccine preventable disease incidence and numbers needed to vaccinate. Vaccine.

[21] Kelly H, Attia J, Andrews R, Heller RF (2004). The number needed to vaccinate (NNV) and population extensions of the NNV: comparison of influenza and pneumococcal vaccine programmes for people aged 65 years and over. Vaccine.

[22] Klugman KP, Madhi SA, Huebner RE, Kohberger R, Mbelle N, Pierce N, Vaccine Trialists Group (2003). A trial of a 9-valent pneumococcal conjugate vaccine in children with and those without HIV infection. N Engl J Med.

[23] Gessner BD, Sutanto A, Linehan M, Djelantik IG, Fletcher T, Gerudug IK, Ingerani, Mercer D, Moniaga V, Moulton LH (2005). Incidences of vaccine-preventable Haemophilus influenzae type b pneumonia and meningitis in Indonesian children: hamlet-randomised vaccine-probe trial. Lancet.

[24] Leino T, Ollgren J, Salo H, Tiihonen P, Kilpi T (2012). First year experience of rotavirus immunisation programme in Finland. Vaccine.

[25] RTS,S Clinical Trials Partnership (2015). Efficacy and safety of RTS, S/AS01 malaria vaccine with or without a booster dose in infants and children in Africa: final results of a phase 3, individually randomised, controlled trial. Lancet.

[26] Barnighausen T, Bloom DE, Cafiero-Fonseca ET, O'Brien JC (2014). Valuing vaccination. Proc Natl Acad Sci U S A.

[27] Bärnighausen T, Oldenburg C, Tugwell P, Bommer C, Ebert C, Barreto M, Djimeu E, Haber N, Waddington H, Rockers P, et al. Quasi-experimental study designs series - Paper 7: assessing the assumptions. J Clin Epidemiol. 2017. doi:10.1016/j.jclinepi.2017.02.017. 10.1016/j.jclinepi.2017.02.017 28365306

[28] Bärnighausen T, Tugwell P, Røttingen J, Shemilt I, Rockers P, Geldsetzer P, Lavis J, Grimshaw J, Daniels K, Brown A et al: Quasi-experimental study designs series - Paper 4: uses and value. J Clin Epidemiol. 2017. doi:10.1016/j.jclinepi.2017.03.012. 10.1016/j.jclinepi.2017.03.012 28365303

[29] Anekwe TD, Newell ML, Tanser F, Pillay D, Barnighausen T (2015). The causal effect of childhood measles vaccination on educational attainment: a mother fixed-effects study in rural South Africa. Vaccine.

[30] Smith LM, Kaufman JS, Strumpf EC, Levesque LE (2015). Effect of human papillomavirus (HPV) vaccination on clinical indicators of sexual behaviour among adolescent girls: the Ontario Grade 8 HPV Vaccine Cohort Study. CMAJ.

[31] Wong K, Campitelli MA, Stukel TA, Kwong JC (2012). Estimating influenza vaccine effectiveness in community-dwelling elderly patients using the instrumental variable analysis method. Arch Intern Med.

[32] Bloom DE (2011). The value of vaccination. Adv Exp Med Biol.

[33] Barnighausen T, Bloom DE, Cafiero ET, O'Brien JC (2013). Valuing the broader benefits of dengue vaccination, with a preliminary application to Brazil. Semin Immunol.

[34] Halloran ME, Longini IM, Struchiner CJ (2010). Design and Analysis of Vaccine Studies.

